# The effectiveness of knee bracing in non‐operative soft tissue and degenerative knee injuries: A systematic review

**DOI:** 10.1002/ksa.70080

**Published:** 2025-09-29

**Authors:** Marc Daniel Bouchard, Justin Gilbert, Michelle Cruickshank, Colin Kruse, Prushoth Vivekanantha, James Yan, Vickas Khanna

**Affiliations:** ^1^ Division of Orthopaedic Surgery McMaster University Hamilton Ontario Canada; ^2^ Michael G. DeGroote School of Medicine McMaster University Hamilton Ontario Canada; ^3^ Division of Health Research Methodology McMaster University Hamilton Ontario Canada; ^4^ Faculty of Health Sciences Education McMaster University Hamilton Ontario Canada; ^5^ Department of Orthopaedic Surgery St. Joseph's Hospital Hamilton Ontario Canada

**Keywords:** degenerative knee injury, knee brace, knee orthosis, non‐operative management, soft tissue knee injury

## Abstract

**Purpose:**

Soft tissue and degenerative knee injuries, including anterior/posterior cruciate ligament (ACL/PCL) injuries, medial knee osteoarthritis (MKOA), and patellofemoral osteoarthritis (PFOA), are common causes of pain and functional decline. Knee bracing is often used as part of non‐operative treatment, but its clinical effectiveness remains uncertain. This systematic review aimed to evaluate the role of bracing in improving pain, function, and preventing surgical conversion in adults with non‐operatively managed knee injuries.

**Methods:**

A systematic review was conducted following PRISMA guidelines. Embase, Ovid MEDLINE, and Ovid Emcare were searched from inception to March 2025. Studies were included if they reported clinical outcomes of knee bracing in adults with soft tissue or degenerative knee injuries treated non‐operatively. Studies focused on biomechanics, prophylaxis, surgery, or paediatric populations were excluded. Data were synthesised narratively with weighted summary statistics.

**Results:**

Seventeen studies (706 patients) were included: six on ACL injuries, three PCL, three MKOA, and five PFOA. MKOA studies showed pooled improvements of +14.6 in KOOS Pain and −1.9 in VAS (SDs 1.0 and 0.5). ACL studies reported a pooled Lysholm gain of +11.8 (SD = 4.3), while PCL outcomes showed large single‐study improvements (KOOS Pain +31.0, IKDC + 30.0). WOMAC scores improved in MKOA (−13.7) but declined in PFOA (−6.4). Failure (surgical conversion) was reported in 13 studies, with the highest rates in PCL (16.4%) and ACL (10.6%), and the lowest in MKOA (0%) and PFOA (4.0%). Complications were infrequently reported; skin irritation was the most common adverse event.

**Conclusion:**

Knee bracing demonstrated the most consistent pain and functional improvements in degenerative and PCL injuries. In contrast, outcomes in ACL injuries were more variable and associated with higher failure rates, underscoring the need for injury‐specific bracing strategies. These findings emphasise the importance of patient selection, brace design, and early intervention, and support the need for higher‐quality studies to guide non‐operative management strategies.

**Level of Evidence:**

Level IV.

**Abbreviations**
ACL: anterior cruciate ligament
IKDC: International Knee Documentation Committee
KOOS: Knee Injury and Osteoarthritis Outcome Score
MKOA: medial knee osteoarthritis
OAK: Osteoarthritis of the Knee score
PCL: posterior cruciate ligament
PFOA: patellofemoral osteoarthritis
PROM: Patient‐Reported Outcome Measure
VAS: Visual Analogue Scale
WOMAC: Western Ontario and McMaster Universities Osteoarthritis Index

## INTRODUCTION

Soft tissue ligamentous injuries, such as those to the anterior cruciate ligament (ACL) and posterior cruciate ligament (PCL), as well as degenerative knee conditions like medial knee osteoarthritis (MKOA) and patellofemoral osteoarthritis (PFOA), are common sources of pain, disability, and functional decline in adults [7, 13, 31]. These conditions are frequently managed conservatively, particularly when mechanical symptoms or significant joint instability do not necessitate surgical intervention [11, 30]. However, in cases of untreated soft tissue injuries, particularly ligamentous tears, inadequate management may predispose patients to long‐term sequelae, including a three‐ to six‐fold increased risk of developing knee osteoarthritis (OA), underscoring the need for effective early intervention strategies [13, 34].

Knee bracing has emerged as a widely used adjunct in the conservative management of soft tissue and degenerative knee conditions, with proposed benefits such as pain reduction, improved function, enhanced proprioception, and slowed disease progression [11, 54]. A variety of bracing designs have been developed to address specific biomechanical deficits. Functional braces are commonly prescribed following ligamentous injuries to restrict abnormal joint motion, reduce mechanical stress, and promote ligament healing and joint stability. These braces are typically characterised by rigid frame designs with hinges and adjustable straps that allow for controlled motion while limiting instability in specific planes [1, 53]. Unloader braces and patellar tracking orthoses are utilised in degenerative conditions such as OA and PFOA to redistribute joint loads and improve joint alignment [6]. Similarly, patellofemoral stabilising braces and infrapatellar straps aim to offload tensile stress on the extensor mechanism in cases of patellofemoral pain or tendinopathy [46]. These devices are intended to facilitate recovery while maintaining patient activity levels and potentially delaying or avoiding surgical intervention [1, 11, 53]. Bracing principles are grounded in biomechanical and proprioceptive mechanisms, aiming to improve joint stability, alignment, and functional performance, while potentially modifying neuromuscular control. Evidence from randomised controlled trials supports these theoretical benefits in select patient populations [35].

Despite the theoretical and biomechanical rationale supporting their use, clinical evidence on the effectiveness of knee braces remains inconsistent [9, 38, 48]. While some studies have demonstrated improvements in pain, stiffness, and functional outcomes, others have reported limited or no additional benefit compared to rehabilitation alone [6, 38, 46, 48]. Such variability may stem from differences in study design, brace type, injury severity, patient populations, and outcome measures employed [9, 48].

Although several reviews have assessed bracing for specific conditions such as ACL injuries or osteoarthritis, no comprehensive synthesis has evaluated the broader role of knee bracing across the spectrum of non‐operative soft tissue and degenerative knee pathologies [11, 38, 48]. By synthesising and analysing the available literature, this systematic review aimed to evaluate the impact of knee bracing on functional outcomes, pain relief, and symptom progression across a broad range of knee pathologies. Ultimately, this study sought to provide evidence‐based guidance to support clinical decision‐making and optimise non‐operative management strategies for patients with soft tissue and degenerative knee injuries.

## METHODS

### Study design and registration

This systematic review was conducted in accordance with the Preferred Reporting Items for Systematic Reviews and Meta‐Analyses (PRISMA) [24] guidelines to ensure transparency and rigour in reporting. The review protocol was registered in PROSPERO [ID: CRD420251021706] prior to the commencement of the review and any amendments were documented and updated accordingly.

### Search strategy and study selection

A comprehensive literature search was conducted across Embase, Ovid MEDLINE, and Ovid Emcare from database inception to March 27, 2025. In addition, the reference lists of all studies meeting inclusion criteria, as well as those of relevant prior systematic reviews, were manually screened to ensure comprehensive article retrieval. The search strategy utilised a combination of Medical Subject Headings (MeSH) and relevant keywords pertaining to knee injuries (e.g., ACL, PCL, MCL, LCL, meniscus, patellofemoral osteoarthritis, patellar tendinopathy and quadriceps tendinopathy) and bracing interventions (e.g., brace, orthosis, strap and sleeve), with Boolean operators used to appropriately combine terms. Full search strategies for each database are detailed in Supporting Information: Table S1.

After duplicate removal, two reviewers (MDB and JG) independently screened all titles and abstracts for relevance. Full‐text articles were retrieved for studies that satisfied inclusion criteria or in cases of uncertainty based on abstract review. Disagreements regarding study selection were resolved through discussion or, when needed, consultation with a third reviewer and senior author (V.K.). Reasons for exclusion at the full‐text stage were documented. Inter‐reviewer agreement was assessed using the kappa (κ) statistic and interpreted using standard thresholds: slight (0.00–0.21), fair (0.21–0.40), moderate (0.41–0.60), substantial (0.61–0.80) and almost perfect agreement (0.81–1.00) [23].

### Eligibility criteria

Studies were eligible for inclusion if they investigated the clinical effectiveness of knee bracing in adult patients (aged 18 years and older) with non‐operatively managed soft tissue or degenerative knee injuries. Relevant conditions from the identified from the search included isolated injuries to the ACL or PCL, medial knee osteoarthritis, and patellofemoral osteoarthritis. Studies were required to evaluate knee bracing, including functional braces, unloader braces, orthoses, sleeves or straps, as part of conservative treatment, either alone or in comparison to non‐bracing interventions.

Eligible study designs included randomised controlled trials (RCTs), prospective or retrospective cohort studies, and case series with at least ten participants. Only studies that reported clinical outcomes such as pain, function, joint stability, symptom progression, or return to activity were included. Secondary outcomes included rates of failure (progression to surgery) and complications. Studies were excluded if they focused on operative management, skeletally immature populations, concomitant or multi‐ligamentous knee injuries, animal or cadaveric models, or purely biomechanical investigations without clinical endpoints. Studies assessing bracing solely for prophylactic use (e.g., injury prevention in uninjured populations) or reporting only kinematic, force plate, or motion analysis data without clinical correlation were also excluded. Articles that were unavailable or not published in English were excluded unless a translated version was available.

### Data extraction

Data extraction was independently conducted by two reviewers (M.D.B. and M.C.) using a standardised data collection form in Microsoft Excel (version 16.90). Extracted variables included study characteristics (author, year of publication, study design, level of evidence, sample size, and follow‐up duration), patient demographics (age, sex and laterality), injury type, brace design, comparator interventions (if applicable), rehabilitation protocols (if reported) and outcome measures (pain scores, patient‐reported outcomes, mobility, complications and progression to surgical intervention). Discrepancies were resolved through discussion and consensus with senior authors.

### Methodological quality assessment

The methodological quality of included studies was assessed by two reviewers (M.D.B. and J.G.) using two tools based on study design. Randomised controlled trials (RCTs) were evaluated using the Cochrane Risk of Bias 2.0 (RoB2) tool, which assesses bias across five domains: randomisation process, deviations from intended interventions, missing outcome data, measurement of the outcome, and selection of the reported result [13]. Each domain was rated as low risk, some concerns, or high risk, with an overall risk of bias judgement assigned accordingly [47].

Non‐randomised studies were independently evaluated by two reviewers (M.D.B. and J.G.) using the Methodological Index for Non‐Randomised Studies (MINORS) criteria. Each study was scored across the relevant MINORS domains, with ideal global scores of 16 for non‐comparative and 24 for comparative studies [45]. For this review, non‐comparative studies were classified as poor (≤8), moderate (9–14) or good quality (15–16), while comparative studies were classified as poor (≤14), moderate (15–22) or good quality (23–24). Any discrepancies in scoring were resolved through consensus with a third senior author (V.K.).

### Data synthesis and analysis

Due to the heterogeneity in study populations, injury types, brace designs, outcome measures, and follow‐up durations, a quantitative meta‐analysis was not feasible. Instead, a narrative synthesis approach was employed. Descriptive statistics were used to summarise study characteristics and outcomes, and findings were organised according to injury type and bracing intervention where possible. Data were summarised using absolute frequencies with corresponding percentages or weighted means with measures of variability (weighted standard deviation or range), as appropriate. Although a quantitative meta‐analysis was not performed, the potential for publication bias was considered. A formal assessment using funnel plots was not feasible; however, publication bias was evaluated qualitatively by reviewing study protocols, trial registries, and examining selective outcome reporting across studies. All analyses were conducted using Microsoft Excel (version 16.90).

## RESULTS

### Search results

**Figure 1:**
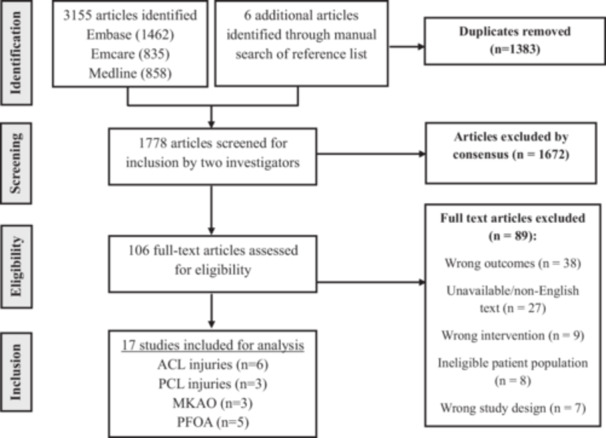
Study selection flowchart based on PRISMA guidelines. ACL, anterior cruciate ligament; MKOA, medial knee osteoarthritis; PCL, posterior cruciate ligament; PFOA, patellofemoral osteoarthritis; PRISMA, Preferred Reporting Items for Systematic Reviews and Meta‐Analyses.

A systematic search of Embase, Ovid Emcare, and Ovid MEDLINE databases identified 3155 potentially relevant studies (Supporting Information: Table S1). An additional six studies were identified through manual screening of reference lists. After removing 1383 duplicates, 1778 unique records remained for title and abstract screening. Agreement between reviewers was substantial at this stage (*κ* = 0.61). Following the exclusion of 1672 articles, 106 full‐text studies were assessed for eligibility, with almost perfect inter‐reviewer agreement at this stage (*κ* = 0.97). A total of 89 full‐text articles were excluded for the following reasons: wrong outcomes (*n* = 38), defined as studies reporting only biomechanical, imaging or kinematic endpoints without clinical or patient‐reported outcomes; non‐English or unavailable full texts (*n* = 27); wrong intervention (*n* = 9), which included studies evaluating orthotics or prophylactic bracing rather than therapeutic knee bracing; ineligible patient populations (*n* = 8), such as paediatric cohorts, post‐operative patients, or individuals with multi‐ligamentous injuries; and inappropriate study design (*n* = 7), including case reports or reviews lacking original data. Ultimately, 17 studies met the inclusion criteria and were included in the final analysis. Among them, six studies focused on ACL injuries, three on PCL injuries, three on medial knee osteoarthritis (MKOA), and five on PFOA (Figure 1).

### Study demographics

Seventeen studies comprising a total of 706 patients were included in this review [2, 4, 5, 8, 20, 21, 22, 25, 28, 32, 33, 34, 40, 41, 43, 49, 52, 55]. Study designs were heterogeneous and included seven randomised controlled trials [5, 8, 28, 41, 43, 49, 52], six prospective cohort studies [2, 4, 20, 32, 33, 55], three prospective case series [21, 22, 40] and one retrospective case series [25]. The level of evidence ranged from I to IV, with six studies meeting criteria for level I evidence [5, 8, 41, 43, 49, 52]. Across all included studies, the weighted mean age of patients was 44.2 years (standard deviation [SD] = 14.4), and the mean follow‐up duration was 11.3 months (SD = 8.1). 54.5% of the patients studied were male (SD = 25.3). The most commonly studied condition was ACL injury, representing 325 patients (46.0%) across six studies [2, 5, 20, 25, 33, 49], followed by PFOA with 235 patients (33.3%, five studies) [8, 27, 43, 52, 55], PCL injury with 88 patients (12.5%, three studies) [21, 22, 40] and MKOA with 58 patients (8.2%, three studies) [4, 32, 41] (Tables 1 and 2).

**Table 1:** Summary of included studies and overall patient demographics.

Author (years)	Study design	Level of evidence	Patients (*n*)	Mean age (range/SD)	Male (%)	Mean follow‐up, months	Injury	Brace type	Duration of bracing (weeks)
Ahn et al. [2], (2010)	PC	III	48	31.8 (19–51)	63.0	21.5	ACL	Hinge (rigid)	8
Beck et al. [4], (2023)	PC	IV	14	43.1 (SD = 9.4)	50.0	3.3	MKOA	Unloader	14.4
Blein‐Ibanez et al. [5], (2024)	RCT	I	36	37.8 (SD = 13.3)	69.4	NR	ACL	Tape	0.57
Callaghan et al. [8], (2015)	RCT	I	63	54.5 (SD = 6.7)	36.5	1.5	PFOA	Functional	6
Jacobi et al. [21], (2010)	PCS	IV	21	29.2 (17–60)	90.5	NR	PCL	Functional	12
Jacobi et al. [20], (2016)	PC	II	86	32 (SD = 14)	61.2	24	ACL	Functional (ACL‐Jack)	16
Jung et al. [22], (2008)	PCS	IV	17	25 (12–56)	NR	NR	PCL	Functional	12
Liu et al. [25], (2019)	RCS	IV	48	26 (SD = 3.63)	100.0	NR	ACL	Tape	1
Merino et al. [28], (2021)^a^	RCT	II	57	64.18 (SD = 7.75)	17.5	12	PFOA	Functional brace, sleeve	52
Ornetti et al. [32], (2015)	PC	IV	20	64.2 (SD = 10.2)	20.0	12	MKOA	Unloader	52
Park et al. [33], (2021)	PC	II	85	35.8 (18–59)	75.3	12	ACL	Hinge functional (unspecified)	12
Rasmussen et al. [40], (2023)	PCS	III	50	35.5 (SD = 10.25)	74.0	NR	PCL	Functional	12
Robert‐Lachaine et al. [41], (2020)	RCT	I	24	57.2 (SD = 8.6)	58.3	NR	MKOA	Functional unloader	36
Shah et al. [43], (2025)	RCT	I	20	55.6 (SD = 3.8)	65.0	1	PFOA	Tape	4
Swirtun et al. [49], (2005)	RCT	I	22	36 (SD = 7.1)	45.4	6	ACL	Functional (SofTec)	12
Yamamoto et al. [52], (2019)	RCT	I	57	64.2 (SD = 7.8)	17.5	3	PFOA	Functional brace, sleeve	12
Zhang et al. [55], (2017)	PC	IV	38	67.2 (SD = 9.0)	23.7	6.5	PFOA	Functional	26
Totals, weighted means/SD	–	–	706	44.2 (SD = 14.4)	54.5 (SD = 25.3)	11.3 (SD = 8.1)	–	11 Functional 4 Unloader 3 Tape 2 Hinge 2 Sleeve	16.1 (SD = 14.5)

Abbreviations: ACL, anterior cruciate ligament; MKOA, medial knee osteoarthritis; NR, not reported; PC, prospective cohort; PCL, posterior cruciate ligament; PCS, prospective case series; PFOA, patellofemoral osteoarthritis; RCT, randomised control trial; RCS, retrospective case series.

a Note that the Merino et al. [28] study was a lesser quality RCT and hence given a level of evidence of II.

When stratified by injury type, patient and intervention characteristics differed notably. PCL cohorts were the youngest, with a weighted mean age of 32.0 years (SD = 4.3) and 78.9% male participants. All three PCL studies used functional braces, though follow‐up duration was either unreported or highly variable. ACL cohorts followed closely, with a mean age of 33.0 years (SD 3.6) and 67.7% male representation. The average follow‐up duration among ACL studies was 17.6 months (SD = 6.4). Bracing modalities in these studies included functional braces (three studies) [20, 24] hinge braces (two studies) [2, 33] and tape‐based interventions (two studies) [5, 25]. In contrast, the MKOA subgroup consisted of an older population with a mean age of 56.2 years (SD 8.0) and 43.1% male representation (SD = 17.1). Bracing interventions in MKOA primarily involved unloader braces (three studies) [4, 32, 41], with one study incorporating a functional brace [41]. Notably, one study [41] evaluated multiple brace types within the same trial, including two distinct unloader designs. The mean follow‐up period for MKOA studies was 8.4 months (SD = 4.3). The PFOA subgroup represented the oldest cohort, with a mean age of 61.3 years (SD = 5.0) and the lowest proportion of male patients at 27.6% (SD = 13.9). Bracing strategies for PFOA included functional braces in four studies [8, 28, 52, 55], patellofemoral sleeves in two studies [28, 52], and taping in one study [43] The average follow‐up for PFOA studies was 5.2 months (SD 4.2) (Table 2).

**Table 2:** Patient demographics and bracing characteristics stratified by injury type.

Author (years)	Study design	Level of evidence	Patients (*n*)	Mean age (range/SD)	Male (%)	Mean follow‐up, months	Injury	Brace	Duration of bracing (weeks)
Ahn et al. [2], (2010)	PC	III	48	31.8 (19–51)	63.0	21.5	ACL	Hinge (rigid)	8
Blein‐Ibanez et al. [5], (2024)	RCT	I	36	37.8 (SD = 13.3)	41.7	NR	ACL	Tape	0.57
Jacobi et al. [20], (2016)	PC	II	86	32 (SD = 14.0)	61.2	24.0	ACL	Functional (ACL‐Jack)	16
Liu et al. [25], (2019)	RCS	IV	48	26 (SD = 3.6)	100.0	NR	ACL	Tape	1
Park et al. [33], (2021)	PC	II	85	35.8 (18–59)	75.3	12.0	ACL	Hinge & functional (unspecified)	12
Swirtun et al. [49], (2005)	RCT	I	22	36 (7.1)	45.4	6.0	ACL	Functional (SofTec)	12
**Totals ACL**	**2 RCT** **3 PC** **1 RCS**	‐	**325**	**33.0 (SD** = **3.6)**	**67.7 (SD** = **17.0)**	**17.6 (SD** = **6.4)**	**6**	**2 Hinge** **3 Functional** **2 Tape**	**9.6 (SD** = **5.8)**
Jacobi et al. [21], (2010)	PCS	IV	21	29.2 (17–60)	90.5	NR	PCL	Functional	12
Jung et al. [22], (2008)	PCS	IV	17	25.0 (12–56)	NR	NR	PCL	Functional	12
Rasmussen et al. [40], (2023)	PCS	III	50	35.5 (SD = 10.3)	74.0	NR	PCL	Functional	12
**Totals PCL**	**3 PCS**	‐	**88**	**32.0 (SD** = **4.3)**	**78.9 (SD** = **7.5)**	‐	**3**	**3 Functional**	**12 (NA)**
Beck et al. [4], (2023)	PC	IV	14	43.1 (SD = 9.4)	50.0	3.3	MKOA	Unloader	14.4
Ornetti et al. [32], (2015)	PC	IV	20	64.2 (SD = 10.2)	20.0	12.0	MKOA	Unloader	52
Robert‐Lachaine et al. [41], (2020)	RCT	I	24	57.2 (SD = 8.6)	58.3	NR	MKOA	Functional, 2xUnloader	36
**Totals MKOA**	**1 RCT** **2 PC**	‐	**58**	**56.2 (SD** = **8.0)**	**43.1 (SD** = **17.1)**	**8.4 (SD** = **4.3)**	**3**	**4 Unloader** **1 Functional**	**36.3 (SD** = **14.2)**
Callaghan et al. [8], (2015)	RCT	I	63	54.5 (SD = 6.7)	36.5	1.5	PFOA	Functional	6
Merino et al. [28], (2021)	RCT	II	57	64.2 (SD = 7.8)	17.5	12.0	PFOA	Functional brace, sleeve	52
Shah et al. [43], (2025)	RCT	I	20	55.6 (SD = 3.8)	65.0	1.0	PFOA	Tape	4
Yamamoto et al. [52], (2019)	RCT	I	57	64.2 (SD = 7.8)	17.5	3.0	PFOA	Functional brace, sleeve	12
Zhang et al. [55], (2017)	PC	IV	38	67.2 (SD = 9.0)	23.7	6.5	PFOA	Functional	26
**Totals PFOA**	**4 RCT** **1 PC**	‐	**235**	**61.3 (SD** = **5.0)**	**27.6 (SD** = **13.9)**	**5.2 (SD** = **4.2)**	**5**	**4 Functional** **2 Sleeves** **1 Tape**	**21.7 (SD** = **18.5)**

Abbreviations: ACL, anterior cruciate ligament; MKOA, medial knee osteoarthritis; NR, not reported; PC, prospective cohort; PCL, posterior cruciate ligament; PCS, prospective case series; PFOA, patellofemoral osteoarthritis; RCS, retrospective case series; RCT, randomised control trial.

Overall, functional bracing was the most frequently reported intervention across all injury types, utilised in 11 studies [8, 20, 21, 22]. This was followed by unloader braces in three studies [4, 32, 41], tape‐based interventions in three studies [5, 25, 43], hinge braces in two studies [2, 33], and patellofemoral sleeves in two studies [28, 52].

Bracing duration was variably reported across studies, with a weighted mean of 16.1 weeks (SD = 14.5). The shortest intervention involved kinesiology tape worn for 4 days (0.57 weeks) [5], while the longest spanned up to 52 weeks in studies of chronic degenerative conditions such as MKOA and PFOA (Table 1) [28, 32]. When stratified by injury type, mean bracing durations were 9.6 weeks (SD = 5.8) for ACL, 12.0 weeks for PCL, 36.3 weeks (SD = 14.2) for MKOA, and 21.7 weeks (SD = 18.5) for PFOA (Table 2).

### Study quality

The methodological quality of included studies varied substantially, reflecting differences in study design and execution. Of the seven randomised controlled trials, three were rated as having low overall risk of bias according to the Cochrane Risk of Bias 2.0 (RoB2) tool (Supporting Information: Table ii) [5, 8, 43]. Two trials demonstrated 'some concerns,' largely due to deviations from intended interventions and issues related to selective outcome reporting [28, 41]. The remaining two studies [49, 52] were classified as high risk of bias, with consistent concerns noted across multiple domains, including inadequate randomisation, insufficient blinding, and incomplete outcome data.

The 10 non‐randomised studies were evaluated using the Methodological Index for Non‐Randomised Studies (MINORS). Most studies were classified as moderate quality, with total scores ranging from 7 to 14 out of a maximum of 16 for non‐comparative designs, and up to 19 out of 24 for the single comparative study [20] Only one study [55] was deemed poor quality, scoring 7 points due to retrospective data collection, unclear follow‐up procedures, and the absence of defined inclusion criteria. Ornetti et al. [32] was the only study classified as high quality, achieving a MINORS score of 14 out of a possible 16 (Supporting Information: Table S3).

No evidence of selective reporting or unpublished negative studies was identified based on available trial registries and study protocols.

### Patient‐reported outcome measures (PROMs)

PROMs were heterogeneously reported across the 17 included studies. The most commonly used instruments were the Knee injury and Osteoarthritis Outcome Score (KOOS) Pain subscale [4, 5, 8, 32, 40, 41], the Western Ontario and McMaster Universities Osteoarthritis Index (WOMAC) [28, 32, 41, 43, 52, 55], and the Visual Analogue Scale (VAS) for pain, reported in six studies each [4, 5, 8, 32, 40, 41, 49]. Functional scoring systems, used primarily in ligament injury cohorts, included the Lysholm Knee Scoring Scale, Tegner Activity Scale, and the International Knee Documentation Committee (IKDC) score. Only one study [22] used the Osteoarthritis of the Knee Score (OAK). Due to variability in outcome measures, reporting formats, and follow‐up durations, PROM results were synthesised descriptively and stratified by injury type and measurement tool (Tables 3 and 4).

**Table 3:** Pre‐ and postoperative patient reported outcome measures across included studies.

Author (years)	Injury	Patients at FU	Lysholm	Tegner	IKDC	KOOS Pain	WOMAC	VAS	OAK
Ahn et al. [2], (2010)	ACL	48	Pre‐int: NR Post‐int: 91.0 (NR)	NR	Pre‐int: NR Post‐int: 91.1 (NR)	NR	NR	NR	NR
Beck et al. [4], (2023)	MKOA	14	NR	NR	NR	Pre‐int: 42.1 (22.7) Post‐int: 64.8 (18.7)	NR	Pre‐int: 5.9 (2.0) Post‐int: 2.0 (1.3)	NR
Blein‐Ibanez et al. [5], (2024)	ACL	36	Pre‐int: 55.5 (7.1) Post‐int: 76.4 (14.1)	NR	NR	Pre‐int: 56.5 (20.2) Post‐int: 65.1 (19.0)	NR	NR	NR
Callaghan et al. [8], (2015)	PFOA	63	NR	NR	NR	Pre‐int: 48.2 (18.4) Post‐int: 57.5 (NR)	NR	Pre‐int: 6.8 (2.1) Post‐int: 5.0 (NR)	NR
Jacobi et al. [21], (2010)	PCL	Initial: 21 12 mo: 21 24 mo: 17	Pre‐int^a^: 98.0 (1.3) 12 mo: 94.0 (5.6) 24 mo: 94.0 (3.3)	Pre‐int^a^: 7.5 (1.3) 12 mo: 7.2 (1.3) 24 mo: 7.2 (1.7)	Pre‐int^a^: 99.0 (1.9) 12 mo: 93.0 (7.5) 24 mo: 95.0 (6.7)	NR	NR	NR	NR
Jacobi et al. [20], (2016)	ACL	66	Pre‐int^a^: 99.7 (1.2) Post‐int: 93.3 (8.3)	Pre‐int^a^: 6.6 (2.0) Post‐int: 5.9 (2.0)	Pre‐int^a^: 96.5 (5.2) Post‐int: 90 (8.7)	NR	NR	NR	NR
Jung et al. [22], (2008)	PCL	17	NR	NR	Pre‐int: NR Post‐int: 90.3 (4.2)	NR	NR	NR	Pre‐int: 68.9 (5.9) Post‐int: 93.4 (6.1)
Liu et al. [25], (2019)	ACL	48	Pre‐int: 76.0 (5.3) Post‐int: 81.0 (3.0)	NR	NR	NR	NR	NR	NR
Merino et al. [28], (2021)	PFOA	38	NR	NR	NR	NR	Pre‐int: 43.0 (18.2) Post‐int: 39.1 (21.6)	NR	NR
Ornetti et al. [32], (2015)	MKOA	18	NR	NR	NR	Pre‐int: 42.6 (12.5) Post‐int: 54.3 (13.2)	Pre‐int: 56.7 (12.8) Post‐int: NR	Pre‐int: 6.3 (1.3) Post‐int: 3.8 (1.7)	NR
Park et al. [33], (2021)	ACL	77	Pre‐int: NR Post‐int: 91.2 (NR)	Pre‐int: 6.9 (NR) Post‐int: 6.2 (NR)	NR	NR	NR	NR	NR
Rasmussen et al. [40], (2023)	PCL	Initial: 50 12 mo: 45 24 mo: 31	NR	NR	Pre‐int: 35.0 (9.7) 12 mo: 61.0 (13.0) 24 mo: 65.0 (13.0)	Pre‐int: 56.0 (24.0) 12 mo: 79.0 (17.0) 24 mo: 87.0 (16.0)	NR	NR	NR
Robert‐Lachaine et al. [41], (2020)	MKOA	21	NR	NR	NR	Pre‐int: 56.7 (4.5) Post‐int: 68.3 (3.3)	Pre‐int: 34.7 (4.3) Post‐int: 21.0 (4.0)	Pre‐int: 2.2 (0.8) Post‐int: 2.2 (0.7)	NR
Shah et al. [43], (2025)	PFOA	20	NR	NR	NR	NR	Pre‐int: 21.0 (3.9) Post‐int:13.9 (3.1)	Pre‐int: 6.0 (1.7) Post‐int: 2.9 (1.0)	NR
Swirtun et al. [49], (2005)	ACL	22	NR	Pre‐int: 7.0 (1.6) Post‐int: NR	NR	NR	NR	Pre‐int: 5.0 (NR) Post‐int: 1.0 (NR)	NR
Yamamoto et al. [52], (2019)	PFOA	57	NR	NR	NR	NR	Pre‐int: 43.3 (16.9) Post‐int: 34.0 (20.1)	NR	NR
Zhang et al. [55], (2017)	PFOA	38	NR	NR	NR	NR	Mean change: −4.0 (NR)	NR	NR

*Note*: All values are presented as mean (SD) where post‐intervention is the last follow‐up point.

Abbreviations: ACL, anterior cruciate ligament; FU, follow‐up; IKDC, International Knee Documentation Committee (0–100 with higher score being better); int, intervention; KOOS Pain, Knee injury and Osteoarthritis Outcome Score (0–100 with higher score being better); Lysholm, Lysholm Knee Scoring Scale (0–100 with higher score being better); MKOA, medial knee osteoarthritis; mo, months (post‐intervention); NR, not reported; OAK, Osteoarthritis of the Knee Score (0–100 with higher score being better); PCL, posterior cruciate ligament; PFOA, patellofemoral osteoarthritis; Tegner, Tegner Activity Scale (0–10 with higher score being better); VAS, Visual Analogue Scale (0–10 with lower score being better); WOMAC, Western Ontario and McMaster Universities Osteoarthritis Index (0–100 with lower score being better).

a These are pre‐injury values whereas the others are post‐injury but pre‐intervention.

**Table 4:** Changes in Patient‐Reported Outcome Measures (PROMs) from baseline to final follow‐up by injury type.

Author (year)	Injury	Patients at FU	Lysholm	Tegner	IKDC	KOOS pain	WOMAC	VAS	OAK
Ahn et al. [2], (2010)	ACL	48	NA	NR	NA	NR	NR	NR	NR
Blein‐Ibanez et al. [5], (2024)	ACL	36	Change: 20.9 Weighted: 752.4	NR	NR	Change: 8.5 Weighted: 306.0	NR	NR	NR
Jacobi et al. [20], (2016)	ACL	66	NA	NA	NA	NR	NR	NR	NR
Liu et al. [25], (2019)	ACL	48	Change: 5.0 Weighted: 240.0	NR	NR	NR	NR	NR	NR
Park et al. [33], (2021)	ACL	77	NA	Change: −0.7 Weighted: −53.9	NR	NR	NR	NR	NR
Swirtun et al. [49], (2005)	ACL	22	NR	NA	NR	NR	NR	Change: −4.0 Weighted: −88.0	NR
**Total ACL**	**6**	**297**	**11.8 (4.3)**	**−0.7 (NA)**	–	**8.5 (NA)**	–	**−4.0 (NA)**	–
Jacobi et al. [21], (2010)	PCL	17	NA	NA	NA	NR	NR	NR	NR
Jung et al. [22], (2008)	PCL	17	NR	NR	NA	NR	NR	NR	Change: 24.5 Weighted: 416.5
Rasmussen et al. [40], (2023)	PCL	31	NR	NR	Change: 30.0 Weighted: 930.0	Change: 31.0 Weighted: 961.0	NR	NR	NR
**Total PCL**	**3**	**65**	–	–	**30.0 (NA)**	**31.0 (NA)**	–	–	**24.5 (NA)**
Beck et al. [4], (2023)	MKOA	14	NR	NR	NR	Change: 22.7 Weighted: 317.8	NR	Change: −3.9 Weighted: −54.6	NR
Ornetti et al. [32], (2015)	MKOA	18	NR	NR	NR	Change: 11.7 Weighted: 210.6	NA	Change: −2.5 Weighted: −45.0	NR
Robert‐Lachaine et al. [41], (2020)	MKOA	21	NR	NR	NR	Change: 11.6 Weighted: 243.6	Change: −13.7 Weighted: −287.7	Change: 0 Weighted: 0	NR
**Total MKOA**	**3**	**53**	–	–	–	**14.6 (1.0)**	**−13.7 (NA)**	**−1.9 (0.5)**	–
Callaghan et al. [8], (2015)	PFOA	63	NR	NR	NR	Change: 9.3 Weighted: 585.9	NR	Change: −1.8 Weighted: −113.4	NR
Merino et al. [28], (2021)	PFOA	38	NR	NR	NR	NR	Change: −3.9 Weighted: −148.2	NR	NR
Shah et al. [43], (2025)	PFOA	20	NR	NR	NR	NR	Change: −7.1 Weighted: −142.0	Change: −3.1 Weighted: −62.0	NR
Yamamoto et al. [52], (2019)	PFOA	57	NR	NR	NR	NR	Change: −9.3 Weighted: −530.1	NR	NR
Zhang et al. [55], (2017)	PFOA	38	NR	NR	NR	NR	Change: −4.0 Weighted: −152.0	NR	NR
**Total PFOA**	**5**	**216**	–	–	–	**9.3 (NA)**	**−6.4 (1.3)**	**−2.1 (0.4)**	–

*Note*: This table summarises the average change in PROM scores from the pre‐intervention period to the final follow‐up across included studies. Where available, both the mean change and the sample size–weighted mean change are provided. Pooled values in the 'Total' rows reflect weighted averages for each outcome within a given injury group. All values are expressed as mean (SD), unless derived from a single study, in which case SD is not reported.

Abbreviations: ACL, anterior cruciate ligament; FU, follow‐up; IKDC, International Knee Documentation Committee (0–100 with higher score being better); KOOS Pain, Knee injury and Osteoarthritis Outcome Score (0–100 with higher score being better); Lysholm, Lysholm Knee Scoring Scale (0–100 with higher score being better); MKOA, medial knee osteoarthritis; NA, not applicable; NR, not reported; OAK, Osteoarthritis of the Knee Score (0–100 with higher score being better); PCL, posterior cruciate ligament; PFOA, patellofemoral osteoarthritis; Tegner, Tegner Activity Scale (0–10 with higher score being better); VAS, Visual Analogue Scale (0–10 with lower score being better); WOMAC, Western Ontario and McMaster Universities Osteoarthritis Index (0–100 with lower score being better).

Pooled changes in PROMs are reported in Table 4, with weighted averages calculated where data were available. For certain studies, PROM change scores were not calculable and are marked as 'NA' in the table. This designation was applied in two scenarios: first, when only a pre‐intervention or post‐intervention score was reported but not both; and second, when the baseline value represented a pre‐injury status rather than a true pre‐intervention baseline. The latter applied to both Jacobi studies [20, 21], which reported functional scores prior to injury onset rather than at the time of brace initiation, thus precluding valid calculation of change from intervention.

### ACL injuries

Across the six studies evaluating ACL injuries, the most consistent improvements were observed in Lysholm and KOOS Pain scores. The pooled mean increase in Lysholm score was +11.8 points (SD = 4.3), calculated from two studies [5, 25], while Tegner activity levels showed a slight decline (−0.7), though this was reported in only one study [33] (Table 4). KOOS Pain improved by a weighted mean of +8.5 points, also based on a single study [5]. VAS scores decreased by 4.0 points in one study [49], indicating potential pain relief, although other ACL studies did not report VAS outcomes. Reporting of IKDC scores was inconsistent across studies.

### PCL injuries

Three studies evaluated PROMs following bracing for isolated PCL injuries [21, 22, 40]. Substantial improvements were reported, though each outcome was based on a single study rather than pooled analysis. Rasmussen et al. [40] demonstrated a 30‐point increase in IKDC score and a 31‐point improvement in KOOS Pain subscale following functional bracing, with outcomes sustained through 24‐month follow‐up (Table 4). Jung et al. [22] reported a 24.5‐point increase in OAK score following a staged immobilisation and bracing protocol. In contrast, Jacobi et al. [21] reported high pre‐injury baseline scores with only minor changes following brace use; likely due to the study utilising pre‐intervention values that reflected pre‐injury function rather than true baseline measurements. WOMAC and VAS outcomes were not reported in any of the included PCL studies.

### MKOA

Three studies assessed PROMs in patients with MKOA [4, 32, 41] KOOS Pain scores improved by a pooled mean of +14.6 points (SD = 1.0) across all studies, with the greatest gains reported in Beck et al. [4]. VAS pain scores were also consistently reported and demonstrated a pooled reduction of −1.9 points (SD = 0.5). In contrast, WOMAC outcomes were reported in only one study [41], which showed a 13.7‐point reduction (Table 4).

### PFOA

Five studies evaluated PROMs in patients with PFOA [8, 28, 43, 52, 55]. The KOOS Pain score was reported in only one study [8] which demonstrated a 9.3‐point improvement. VAS pain scores were reported in two studies [8, 43] and showed a pooled mean reduction of 2.1 points (SD = 0.4). WOMAC outcomes were more heterogeneous, with a pooled mean change of −6.4 points (SD = 1.3) across four studies [28, 43, 52, 55], reflecting overall functional benefit. This trend was driven primarily by findings from Merino et al. [28] and Yamamoto et al. [52]. No PFOA studies reported Lysholm, Tegner, IKDC, or OAK scores (Tables 3 and 4).

### Failure rates

Nine of the 17 included studies reported failure rates, which was defined as progression to surgical intervention following initial non‐operative bracing treatment. Studies that did not report or comment on failure were excluded from pooled failure rate calculations. Across the nine studies, a total of 441 patients were assessed, with 42 patients ultimately undergoing surgical intervention, corresponding to an overall pooled failure rate of 9.5% (Table 5).

**Table 5:** Reported failure rates across included studies.

Author (years)	Injury	Brace type	Failure rate (*n*)	Failure rate (%)
Ahn et al. [2], (2010)	ACL	Hinge	1/48	2.1
Beck et al. [4], (2023)	MKOA	Unloader	NR	NR
Blein‐Ibanez et al. [5], (2024)	ACL	Tape	0/36	0
Callaghan et al. [8], (2015)	PFOA	Functional	0/63	0
Jacobi et al. [21], (2010)	PCL	Functional	NR	NR
Jacobi et al. [20], (2016)	ACL	Functional	18/86	20.9
Jung et al. [22], (2008)	PCL	Functional	4/17	23.5
Liu et al. [25], (2019)	ACL	Tape	NR	NR
Merino et al. [28], (2021)	PFOA	Functional Brace, Sleeve	NR	NR
Ornetti et al. [32], (2015)	MKOA	Unloader	0/18	0
Park et al. [33], (2021)	ACL	Hinge Functional	8/85	9.4
Rasmussen et al. [40], (2023)	PCL	Functional	7/50	14.0
Robert‐Lachaine et al. [41], (2020)	MKOA	Functional Unloader	NR	NR
Shah et al. [43], (2025)	PFOA	Tape	NR	NR
Swirtun et al. [49], (2005)	ACL	Functional	NR	NR
Yamamoto et al. [52], (2019)	PFOA	Functional Brace, Sleeve	NR	NR
Zhang et al. [55], (2017)	PFOA	Functional	4/38	10.5
**Totals**	**6 ACL** **3 PCL** **3 MKOA** **5 PFOA**	**11 Functional** **4 Unloader** **3 Tape** **2 Hinge** **2 Sleeve**	**42/441**	**9.5%**

*Note*: Failure, defined as progression to surgery, is summarised by study, injury type, and brace classification.

Abbreviations: ACL, anterior cruciate ligament; MKOA, medial knee osteoarthritis; NR, not reported; PCL, posterior cruciate ligament; PFOA, patellofemoral osteoarthritis.

### Failure rates by injury type

When stratified by injury type, failure rates were highest among ligamentous injuries. In the ACL subgroup, four studies [2, 5, 20, 33] reported failure rates, with a pooled rate of 10.6% (27/255 patients). The highest individual rate was observed in Jacobi et al. [20], where 20.9% of patients treated with a functional brace progressed to surgery. In contrast, Blein‐Ibáñez et al. [5] reported no surgical conversions among patients treated with tape‐based interventions.

In the PCL subgroup, two of the three studies reported failure [22, 40], with a pooled rate of 16.4% (11/67 patients). All PCL studies used functional braces.

For MKOA, only one study [32] reported surgical conversion, with zero patients progressing to surgery (0/18). Although three studies investigated bracing for MKOA, the other two [4, 41] did not report failure data. As such, the pooled failure rate for MKOA was 0% (Table 6).

**Table 6:** Failure rates stratified by injury type.

Author (years)	Failure rate (*n*)	Failure rate (%)	Injury	Brace
Ahn et al. [2], (2010)	1/48	2.1	ACL	Hinge
Blein‐Ibanez et al. [5], (2024)	0/36	0	ACL	Tape
Jacobi et al. [20], (2016)	18/86	20.9	ACL	Functional
Liu et al. [25], (2019)	NR	NR	ACL	Tape
Park et al. [33], (2021)	8/85	9.4	ACL	Hinge & Functional
Swirtun et al. [49], (2005)	NR	NR	ACL	Functional
**Totals ACL**	**27/255**	**10.6**	**6**	**2 Hinge** **3 Functional** **2 Tape**
Jacobi et al. [21], (2010)	NR	NR	PCL	Functional
Jung et al. [22], (2008)	4/17	23.5	PCL	Functional
Rasmussen et al. [40], (2023)	7/50	14.0	PCL	Functional
**Totals PCL**	**11/67**	**16.4**	**3**	**3 Functional**
Beck et al. [4], (2023)	NR	NR	MKOA	Unloader
Ornetti et al. [32], (2015)	0/18	0	MKOA	Unloader
Robert‐Lachaine et al. [41], (2020)	NR	NR	MKOA	Functional, 2xUnloader
**Totals MKOA**	**0/18**	**0**	**3**	**4 Unloader** **1 Functional**
Callaghan et al. [8], (2015)	0/63	0	PFOA	Functional
Merino et al. [28], (2021)	NR	NR	PFOA	Functional Brace, Sleeve
Shah et al. [43], (2025)	NR	NR	PFOA	Tape
Yamamoto et al. [52], (2019)	NR	NR	PFOA	Functional Brace, Sleeve
Zhang et al. [55], (2017)	4/38	10.5	PFOA	Functional
**Totals PFOA**	**4/101**	**4.0**	**5**	**4 Functional** **2 Sleeves** **1 Tape**

*Note*: Failure rates are summarised for each injury subtype (ACL, PCL, MKOA and PFOA). Only studies reporting failure were included in total rate calculations.

Abbreviations: ACL, anterior cruciate ligament; MKOA, medial knee osteoarthritis; NR, not reported; PCL, posterior cruciate ligament; PFOA, patellofemoral osteoarthritis.

In the PFOA subgroup, five studies were included, but only two reported progression to surgery. Zhang et al. [55] was the only study to report surgical conversion, with a rate of 10.5% (4/38). Callaghan et al. [8] also reported zero failures among 63 patients treated with a functional brace. The remaining studies [28, 43, 52] did not report surgical outcomes, limiting pooled analysis. The calculated failure rate for PFOA was 4.0% (4/101), the lowest among the four subgroups evaluated (Table 6).

Further descriptive analysis of the nine studies that reported failure outcomes is summarised in Table 7. Most studies defined failure as persistent instability, progression in clinical tests (e.g., Lachman or pivot shift ≥ grade 2) or unrelieved pain. These studies varied in the timing of brace initiation, with a weighted mean bracing duration of 13.4 weeks (SD = 10.1) and a mean time to surgery of 13.9 months (SD = 6.2) among those who progressed to operative treatment (Table 7).

**Table 7:** Timing of bracing and surgical conversion among studies reporting failure outcomes.

Author (year)	Injury	Population size	Timing of brace intervention	Mean time to surgery (months)	Failure rate/surgical conversion	Criteria for surgery/failure definition	Notes
Ahn et al. [2], (2010)	ACL	48	8	21.5	1/48 (2.1%)	Progression to Lachman/pivot shift ≥ Grade 2	Strict inclusion led to excellent outcomes
Blein‐Ibanz et al. [5], (2024)	ACL	36	0.57	NA	0/36	NA	Taping, not bracing. Very short intervention period (4 days)
Callaghan et al. [8], (2015)	PFOA	63	6	NA	0/63	NA	High patient‐reported brace compliance
Jacobi et al. [20], (2016)	ACL	86	16	NR	18/86 (20.9%)	Persistent instability or recurrent symptoms	Higher failure in young active males
Jung et al. [22], (2008)	PCL	17	12	NR	4/17 (23.5%)	Grade II–III posterior drawer test with pain and/or instability	Missed diagnosis or under‐recognised instability patterns may contribute to failure
Ornetti et al. [32], (2015)	MKOA	18	52	NA	0/18	NA	Careful selection of patients with minimal prior indication for surgery
Park et al. [33], (2021)	ACL	85	12	NR	8/85 (9.4%)	Progression to Lachman/pivot shift ≥ Grade 2	Early intervention ( < 2 weeks) had better success
Rasmussen et al. [40], (2023)	PCL	50	12	12.5	7/50 (14.0%)	NR	2 isolated PCL injuries; 5 knee dislocation injuries
Zhang et al. [55], (2017)	PFOA	38	26	6.0	4/38 (10.5%)	Pain/dissatisfaction	Patients converted to joint arthroplasty
**Totals**	**4 ACL** **2 PCL** **1 MKOA** **2 PFOA**	**441**	**13.4 (SD** = **10.1)**	**13.9 (6.2)**	**42/441 (9.5%)**	**–**	**–**

Abbreviations: ACL, anterior cruciate ligament; MKOA, medial knee osteoarthritis; NR, not reported; PCL, posterior cruciate ligament; PFOA, patellofemoral osteoarthritis; SD, standard deviation.

### Complications

Only three studies [20, 22, 32] in this review explicitly reported complications associated with knee bracing, encompassing a combined total of 102 patients. Across these studies, 21 complications were identified, corresponding to an overall complication rate of 20.6%.

Jacobi et al. [20] evaluated the *ACL‐Jack brace*, a dynamic posterior drawer brace used for non‐operative management of acute ACL injuries. In this cohort of 66 patients, 11 complications were reported (16.7%), including 10 cases of skin irritation and one case of arthrofibrosis.

Ornetti et al. [32] examined the *OdrA distraction‐rotation valgus knee brace*, a custom unloader brace designed for patients with medial knee osteoarthritis. Among 19 patients, seven complications were reported (36.8%), including six cases of skin irritation and one case of worsening varicose veins.

Jung et al. [22] studied a two‐stage protocol for *posterior cruciate ligament (PCL) injuries*, in which patients were initially immobilised in a cylinder cast followed by functional bracing. In this group of 17 patients, three cases (17.6%) of complications were reported, all of which were related to adverse effects from prolonged immobilisation.

Overall, skin irritation or breakdown was the most frequently reported complication, occurring in 16 patients (15.7%), primarily among those using the ACL‐Jack brace or the OdrA distraction‐rotation valgus knee brace. Other less common complications included immobilisation‐related sequelae (*n* = 3) [22], and single reports of arthrofibrosis [20] and varicose vein exacerbation [32], each observed in separate studies. No studies reported brace‐related mechanical failures or neurovascular complications (Table 8).

**Table 8:** Summary of brace‐related complications.

Complications	ACL‐Jack Brace (*n* = 66)	OdrA distraction‐rotation valgus knee brace (*n* = 19)	Cylinder cast then PCL brace (*n* = 17)	Total cases (*n* = 102)
Skin lesions	10	6	0	16
Arthrofibrosis	1	0	0	1
Varicose vein worsening	0	1	0	1
Long term immobilisation	0	0	3	3
Interference screw shortened	0	0	0	0
**Total**	**11 (16.7%)**	**7 (36.8%)**	**3 (17.6%)**	**21 (20.6%)**

Abbreviations: ACL, anterior cruciate ligament; OdrA, osteoarthritis (OA) distraction‐rotation orthotic device; PCL, posterior cruciate ligament.

## DISCUSSION

This systematic review evaluated the clinical effectiveness of knee bracing across a range of non‐operatively managed soft tissue and degenerative knee conditions, including ACL and PCL injuries, MKOA, and PFOA. Across 17 studies and 706 patients, bracing was generally associated with improvements in pain and function, though the magnitude and consistency of benefit varied substantially by injury type, brace design, and study quality.

PROMs demonstrated improvements across most injury subgroups, though the ability to conduct pooled analyses was restricted by inconsistent reporting. This was particularly evident in ACL and PCL studies, where PROMs were often reported in only a single study, limiting the generalisability of findings. Conversely, MKOA and PFOA studies allowed for limited pooling of KOOS Pain, VAS and WOMAC scores.

In ACL injuries, the pooled Lysholm score improved by +11.8 points, exceeding the reported MCID range of 4.8 points following ACL injury [12] and 9.9 points after ACL reconstruction [29], suggesting a clinically meaningful enhancement in knee function [27]. In contrast, the Tegner activity score declined by −0.7 points, which falls below the MCID threshold of ~1.0 points [37, 50]. This minimal change, drawn from a single study [49], likely reflects preservation of activity rather than meaningful recovery. KOOS Pain (+8.5) and VAS pain (−4.0) scores also surpassed MCID thresholds in the literature (6–8 [3, 42] and 1.5–2.0 [10, 18], respectively), but both were likewise derived from single studies, limiting generalisability. Additionally, Jacobi et al. [20] reported pre‐injury rather than pre‐intervention baseline scores, while Park et al. [33] provided only post‐intervention values. This limited the ability to quantify change attributable to bracing and reduced the interpretability of treatment effects.

PCL studies showed the greatest numerical improvement, although all results were based on single studies. Rasmussen et al. [40] reported scores of +30.0 in IKDC and +31.0 in KOOS Pain, far exceeding MCIDs of 10–16 [19, 34] and 8–10 [15, 37], respectively. Jung et al. [22] noted a + 24.5‐point improvement in OAK score, though a validated MCID for OAK is not established. While the reliance on single studies limits generalisability, the magnitude of these improvements suggests that bracing may provide meaningful symptomatic benefit in select patients with isolated PCL injuries, underscoring the need for further validation in well‐designed, comparative trials.

MKOA studies allowed for greater pooling of data, with KOOS Pain improving by +14.6 points (SD = 1.0) and VAS decreasing by −1.9 (SD = 0.5), both surpassing MCIDs and supporting the clinical effectiveness of bracing [14, 15, 37]. WOMAC scores, reported in only one study [41], improved by 13.7 points, also exceeding the MCID of 9–12 points [16, 26], but could not be pooled. Interestingly, this study demonstrated no change in VAS, despite improvement in function. This discrepancy may reflect the short follow‐up period, crossover design, or limited baseline symptom severity.

In PFOA, pooled VAS scores from two studies [8, 43] showed a 2.1‐point reduction (SD = 0.4), indicating a modest but consistent analgesic effect. KOOS Pain improved by 9.3 points in one study [8], exceeding MCID thresholds, while WOMAC outcomes were more heterogeneous. The pooled WOMAC score declined by 6.4 points (SD = 1.3), driven largely by Yamamoto et al. [52], suggesting that while pain may improve with bracing in PFOA, functional outcomes remain variable and may depend on brace design, alignment correction, or adherence.

Failure, defined as progression to surgery, was reported in nine studies with an overall pooled rate of 9.5%. The highest failure rates were observed in PCL (16.4%) and ACL (10.6%) cohorts, likely reflecting the acute nature and mechanical demands of ligamentous injuries [17, 39, 51]. These injuries often result in functional instability that bracing alone may be insufficient to control, particularly in younger or high‐demand individuals [39, 51]. This trend is consistent with prior literature; the KANON trial reported that 51% of ACL‐injured patients initially managed with structured rehabilitation ultimately underwent delayed surgical reconstruction within 5 years [17]. Other cohort studies have documented even higher surgical conversion rates, with up to 60% of non‐operatively treated ACL injuries progressing to surgery, and failure rates approaching 89% among individuals under 25 or those participating in pivoting sports [51].

Timing of brace initiation and patient selection may further explain variability in failure rates across studies. Notably, earlier brace application appeared to correlate with better outcomes in select cases. For example, Park et al. [33] observed improved non‐operative success when bracing was initiated within 2 weeks of injury. In contrast, Jacobi et al. [20] reported higher failure rates among younger, more active individuals, particularly when bracing was delayed or applied after prolonged conservative treatment. These findings suggest that both early intervention and appropriate patient selection are key determinants of bracing success.

By contrast, degenerative conditions such as MKOA and PFOA demonstrated substantially lower failure rates in this review (0% and 4.0%, respectively). This likely reflects both the gradual progression of these conditions and the suitability of bracing as a temporising or symptom‐modifying intervention [44]. Supporting this, longitudinal studies of medial knee OA have shown that the majority of patients managed with unloader bracing or comprehensive conservative therapy are able to delay or avoid surgery for at least two years, with some cohorts reporting no surgical conversions over extended follow‐up periods [44]. These trends align with broader clinical observations that surgical crossover is significantly more common in acute ligament injuries than in chronic degenerative knee conditions.

Brace design may also influence failure rates. Higher rates of failure were observed in studies using functional or hinged braces, while tape‐based interventions demonstrated more favourable outcomes. For instance, Jacobi et al. [20] reported a 20.9% surgical conversion rate with a dynamic functional brace, whereas Blein‐Ibáñez et al. [5] observed no surgical conversions in patients treated with tape‐based bracing. These findings align with prior literature suggesting that functional bracing may be insufficient to restore dynamic stability in high‐demand individuals, particularly those returning to pivoting sports [53]. It is also possible that injury severity influenced brace selection. Less severe injuries, such as partial ligament tears, were more likely managed with taping, while full‐thickness tears or functionally unstable knees were more likely treated with rigid functional bracing. The influence of patient‐specific factors further complicates outcome interpretation. In the Park et al. study [33], 31 ACL patients had co‐existing meniscal pathology with 10 of them requiring surgery, highlighting that brace failure may be confounded by unaddressed intra‐articular lesions. This underscores the importance of appropriate patient selection and thorough clinical and imaging evaluation before initiating non‐operative bracing protocols. In contrast to ligamentous injuries, failure rates in degenerative conditions such as MKOA and PFOA were markedly lower in this review (0% and 4.0%, respectively), supporting the role of unloader and patellofemoral‐specific braces as symptom‐modifying tools in these populations [44]. However, reporting of surgical conversions was often incomplete or inconsistent, limiting confidence in the pooled estimates.

Complications were infrequently reported, with only three studies detailing adverse events. The overall complication rate was 20.6%, with skin irritation accounting for the majority of cases. Notably, no brace‐related mechanical failures or neurovascular complications were reported, suggesting that knee bracing is generally safe when properly fitted. Nonetheless, underreporting is likely, and future studies should systematically evaluate comfort, adherence, and skin‐related side effects.

Several limitations of this review must be acknowledged. First, the heterogeneity in study design, injury chronicity, and brace type precluded meta‐analysis for most outcomes. Therefore, a formal certainty‐of‐evidence assessment (e.g., using the GRADE approach) could not be adequately performed. Many PROMs were reported in only one study per injury type, particularly in ACL and PCL cohorts, limiting the generalisability of MCID‐based interpretations. Even in MKOA and PFOA, where pooling was feasible for KOOS Pain, VAS and WOMAC, the overall number of studies contributing to each analysis remained small. Second, the inconsistent use of baseline definitions, particularly pre‐injury versus pre‐intervention scores, hindered direct comparisons of treatment effect. Third, surgical progression was not uniformly defined or consistently reported, and confounding factors such as concomitant meniscal pathology may have influenced failure rates. The timing of brace initiation (i.e., early versus delayed application) was also variably reported and could not be reliably analysed, although it may influence outcomes such as joint stability, proprioception, and neuromuscular recovery. Brace selection also reflected expected demographic trends where functional bracing predominated in ACL and PCL injuries and unloader and sleeve‐based interventions were favoured in MKOA and PFOA. However, few studies directly compared brace types or stratified outcomes accordingly. Moreover, the ‘functional’ brace category encompassed devices with distinct biomechanical profiles, ranging from passive hinged supports to dynamic load‐modifying braces, yet insufficient reporting precluded subgroup analysis by brace design. Fourth, most included studies did not report or control for concurrent rehabilitation protocols, such as quadriceps strengthening or proprioceptive training, which may have contributed to the observed improvements. This co‐intervention bias limits the ability to isolate the effect of bracing alone. Nevertheless, bracing is typically used as an adjunct to structured rehabilitation, and its clinical effectiveness should be interpreted within the context of multimodal treatment strategies. Recent consensus statements, such as the ESSKA Meniscus Rehabilitation Consensus, emphasise individualised, phase‐based rehabilitation protocols that may include bracing as part of a comprehensive, conservative approach [36]. Finally, complications were likely underreported, and most studies did not track adherence or compliance to bracing protocols, including daily wear duration, intensity, or whether bracing was continuous versus activity‐specific. This limited the ability to evaluate real‐world effectiveness and external validity.

Future studies should prioritise prospective randomised designs with standardised outcome measures, baseline definitions, and MCID‐based reporting to enable more robust comparisons. Comparative trials stratified by brace type, injury pattern, and patient characteristics will be essential to guide individualised, evidence‐based bracing strategies.

## CONCLUSION

Knee bracing may offer modest pain relief and functional benefit across a spectrum of non‐operatively managed knee injuries, with the most consistent improvements observed in degenerative conditions and PCL injuries. While PROMs frequently met MCID thresholds and complication rates were low, findings should be interpreted with caution given the limited quality and consistency of available evidence, especially in ACL injuries where outcomes were more variable. Identifying appropriate indications for bracing may not only improve patient outcomes but also help justify reimbursement from government health systems and private insurers, particularly given the high cost of certain brace designs. Further high‐quality studies are needed to clarify which brace types are most effective for specific injury patterns and patient populations.

## AUTHOR CONTRIBUTIONS

All authors contributed to study design, data collection, analysis, and manuscript preparation.

## CONFLICTS OF INTEREST STATEMENT

Dr. Vickas Khanna reports the following disclosures: paid consultant for Stryker Canada and Zimmer Biomet Canada; received speaker fees from Sanofi and Bioventus; and received travel fees from Ossur. The remaining authors declare no conflicts of interest.

## ETHICS STATEMENT

The review protocol was registered in PROSPERO [ID: CRD420251021706].

## Supporting information

Supporting information.

Supporting information.

Supporting information.

Supporting information.

## Data Availability

The data that supports the findings of this study are available in the supplementary material of this article.
