# Supplementary material for: The effectiveness of knee bracing in non‐operative soft tissue and degenerative knee injuries: A systematic review
Source: Knee Surg Sports Traumatol Arthrosc. 2025 Sep 29;33(12):4446–65. doi: 10.1002/ksa.70080 (PMC12684342; doi:10.1002/ksa.70080)
Supplement: Supplementary file 2 — Supporting information. [file KSA-33-4446-s004.docx]

**Supplementary Table i).** MeSH terms and Boolean operators used to develop search strategy from Embase, Emcare and OVID Medline databases.

**Database: Embase <1974 to 2025 March 27>**
**Search Strategy:**

1. Anterior cruciate ligament.mp. or exp anterior cruciate ligament/ or ACL.mp. (48304)
2. (Meniscus or meniscal).mp. or exp knee meniscus/ (32084)
3. ("Lateral collateral ligament" or LCL).mp or ("Medial collateral ligament" or MCL).mp. (36501)
4. exp posterior cruciate ligament/ or ("Posterior cruciate ligament" or PCL).mp (25611)
5. exp Chondromalacia Patellae/ or "Chondromalacia patella*".mp (1102)
6. ("Patellofemoral osteoarthritis" or PFOA).mp (6632)
7. ("Patellar tendinopathy" or "Jumper's knee").mp (1128)
8. "Quadriceps tendinopathy".mp (30)
9. 1 or 2 or 3 or 4 or 5 or 6 or 7 or 8(136615)
10. brace.ti,ab or exp knee brace/ (9572)
11. orthosis.ti,ab or exp knee orthosis/ (8403)
12. strap.ti,ab (2890)
13. sleeve.ti,ab (30688)
14. 10 or 11 or 12 or 13 (48936)
15. 9 and 14 (1462)

**Database: Ovid MEDLINE(R) ALL <1946 to March 27, 2025>**
**Search Strategy:**

1. Anterior cruciate ligament.mp. or exp Anterior Cruciate Ligament/ or ACL.mp. (35792)
2. (Meniscus or meniscal).mp. or exp Tibial Meniscus Injuries/ or exp Meniscus/ (22736)
3. ("Lateral collateral ligament" or LCL).mp or ("Medial collateral ligament" or MCL).mp. (52784)
4. exp Posterior Cruciate Ligament/ or ("Posterior cruciate ligament" or PCL).mp (21125)
5. exp Chondromalacia Patellae/ or "Chondromalacia patella*".mp (458)
6. ("Patellofemoral osteoarthritis" or PFOA).mp (5921)
7. ("Patellar tendinopathy" or "Jumper's knee").mp (868)
8. "Quadriceps tendinopathy".mp (18)
9. 1 or 2 or 3 or 4 or 5 or 6 or 8 (129775)
10. brace.ti,ab or exp Braces/ (10195)
11. orthosis.ti,ab or exp Orthotic Devices/ (19721)
12. strap.ti,ab (2177)
13. sleeve.ti,ab (16651)
14. 10 or 11 or 12 or 13 (42199)
15. 9 and 14 (858)

**Database: Ovid Emcare <1995 to 2024 Week 13>**
**Search Strategy:**

1. Anterior cruciate ligament.mp. or exp anterior cruciate ligament/ or ACL.mp. (24770)
2. (Meniscus or meniscal).mp. or exp knee meniscus/ (13750)
3. ("Lateral collateral ligament" or LCL).mp or ("Medial collateral ligament" or MCL).mp. (5967)
4. exp posterior cruciate ligament/ or ("Posterior cruciate ligament" or PCL).mp (7584)
5. exp Chondromalacia Patellae/ or "Chondromalacia patella*".mp (407)
6. ("Patellofemoral osteoarthritis" or PFOA).mp (781)
7. ("Patellar tendinopathy" or "Jumper's knee").mp (739)
8. "Quadriceps tendinopathy".mp (14)
9. 1 or 2 or 3 or 4 or 5 or 6 or 7 or 8 (46190)
10. brace.ti,ab or exp knee brace/ (4733)
11. orthosis.ti,ab or exp knee orthosis/ (4504)
12. strap.ti,ab (903)
13. sleeve.ti,ab (4921)
14. 10 or 11 or 12 or 13 (13600)
15. 9 and 14 (835)
