# Supplementary material for: The effectiveness of knee bracing in non‐operative soft tissue and degenerative knee injuries: A systematic review
Source: Knee Surg Sports Traumatol Arthrosc. 2025 Sep 29;33(12):4446–65. doi: 10.1002/ksa.70080 (PMC12684342; doi:10.1002/ksa.70080)
Supplement: Supplementary file 3 — Supporting information. [file KSA-33-4446-s003.docx]

**Supplementary Table ii).** Risk of Bias Assessment for Randomized Controlled Trials Using the RoB 2.0 Tool.

| **Study** | **Randomization Process** | **Deviations from Intended Interventions** | **Missing Outcome Data** | **Measurement of Outcome** | **Selection of Reported Result** | **Overall Risk of Bias** |
| --- | --- | --- | --- | --- | --- | --- |
| **Blein-Ibáñez (2024) [5]** | Low | Low | Low | Low | Low | Low |
| **Callaghan (2015) [8]** | Low | Low | Low | Low | Low | Low |
| **Merino (2021) [27]** | Some concerns | Some concerns | Low | Low | Low | Some concerns |
| **Robert-Lachaine (2020) [38]** | Low | Some concerns | Low | Low | Some concerns | Some concerns |
| **Shah (2022) [39]** | Low | Low | Low | Low | Low | Low |
| **Swirtun (2005) [45]** | Some concerns | Some concerns | Low | Some concerns | Some concerns | High |
| **Yamamoto (2019) [47]** | Some concerns | Some concerns | Some concerns | Low | Some concerns | High |

**
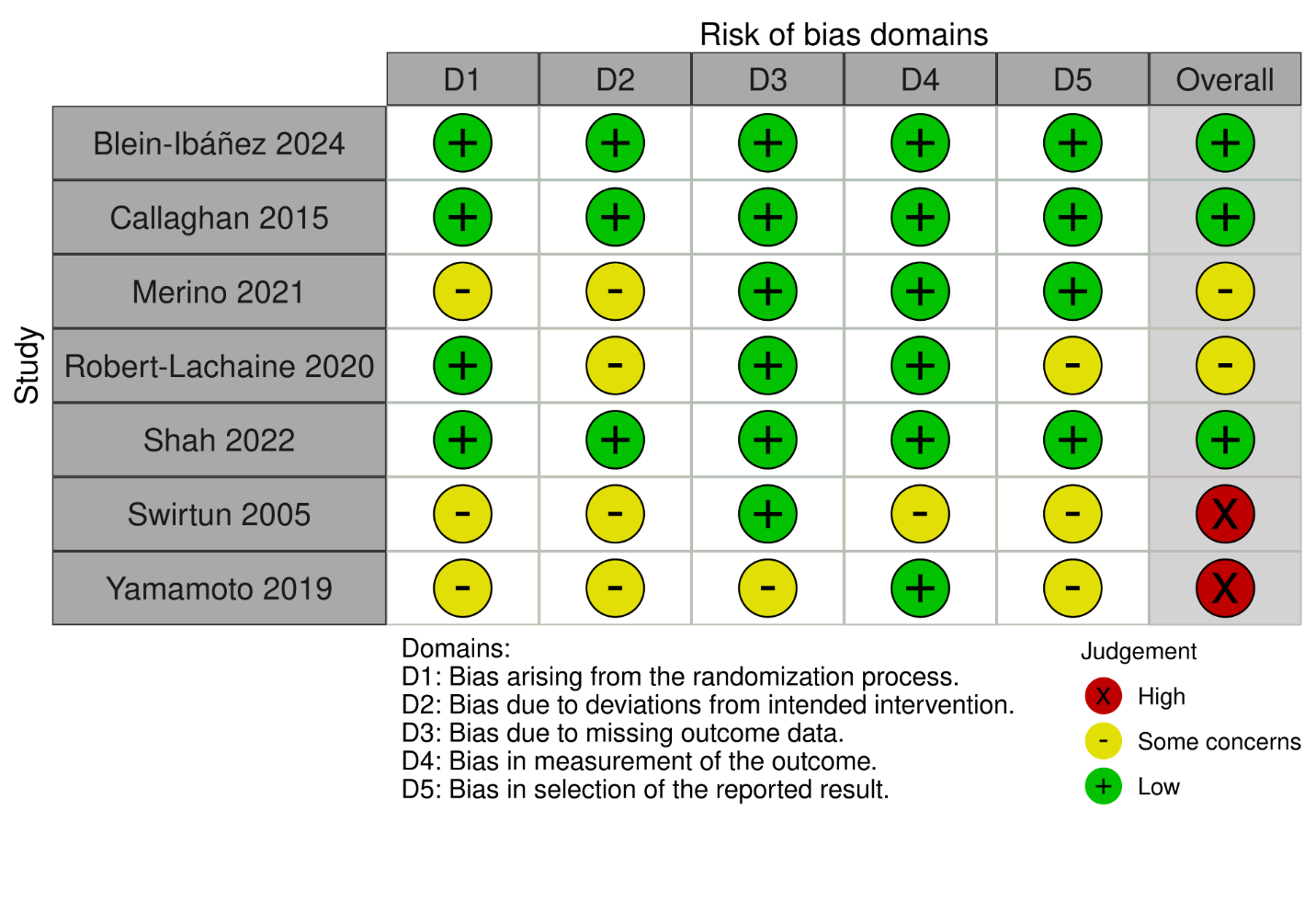
**
