# Supplementary material for: The effectiveness of knee bracing in non‐operative soft tissue and degenerative knee injuries: A systematic review
Source: Knee Surg Sports Traumatol Arthrosc. 2025 Sep 29;33(12):4446–65. doi: 10.1002/ksa.70080 (PMC12684342; doi:10.1002/ksa.70080)
Supplement: Supplementary file 4 — Supporting information. [file KSA-33-4446-s002.docx]

**Supplementary Table iii).** MINORS score calculations quality assessment of all included papers in review.

| Criteria | *Ahn (2010) [2]* | *Beck (2023) [4]* | *Jacobi (2010) [21]* | *Jacobi (2016) [20]* | *Jung (2008) [22]* | *Liu (2019) [25]* | *Ornetti (2015) [32]* | *Park (2021) [33]* | *Rasmussen (2023) [40]* | *Zhang (2017) [55]* |
| --- | --- | --- | --- | --- | --- | --- | --- | --- | --- | --- |
| 1. A clearly stated aim | 2 | 2 | 2 | 2 | 2 | 2 | 2 | 2 | 2 | 2 |
| 2. Inclusion of consecutive patients | 1 | 1 | 1 | 1 | 1 | 1 | 2 | 2 | 2 | 0 |
| 3. Prospective collection of data | 0 | 2 | 0 | 2 | 2 | 0 | 2 | 2 | 2 | 0 |
| 4. Endpoints appropriate to the aim of study | 2 | 2 | 2 | 2 | 2 | 2 | 2 | 2 | 2 | 2 |
| 5. Unbiased assessment of study endpoint | 1 | 1 | 0 | 1 | 1 | 1 | 1 | 1 | 1 | 1 |
| 6. Follow-up period appropriate for the aim of study | 2 | 1 | 2 | 2 | 2 | 1 | 2 | 2 | 2 | 2 |
| 7. Loss to follow-up less than 5% | 2 | 1 | 2 | 2 | 2 | 2 | 1 | 1 | 0 | 0 |
| 8. Prospective calculation of study size | 0 | 0 | 0 | 0 | 0 | 0 | 2 | 0 | 1 | 0 |
|  |  |  |  |  |  |  |  |  |  |  |
| 9. An adequate  control group |  |  |  | 2 |  |  |  |  |  |  |
| 10. Contemporary  groups |  |  |  | 2 |  |  |  |  |  |  |
| 11. Baseline  equivalence of  groups |  |  |  | 1 |  |  |  |  |  |  |
| 12. Adequate  statistical  analyses |  |  |  | 2 |  |  |  |  |  |  |
| **TOTAL MINORS SCORE** | **10** | **10** | **11** | **19** | **12** | **9** | **14** | **12** | **12** | **7** |
| **Maximum possible score** | **16** | **16** | **16** | **24** | **16** | **16** | **16** | **16** | **16** | **16** |

**Legend (Total MINORS Score)** poor quality; moderate quality; good quality

*MINORS = Methodological Index for Non-randomized Studies*
